# Rhinovirus Induces Basolateral Release of IL-17C in Highly Differentiated Airway Epithelial Cells

**DOI:** 10.3389/fcimb.2020.00103

**Published:** 2020-03-13

**Authors:** Kyla C. Jamieson, Shahina Wiehler, Aubrey N. Michi, David Proud

**Affiliations:** Department of Physiology & Pharmacology, Cumming School of Medicine, Snyder Institute for Chronic Diseases, University of Calgary, Calgary, AB, Canada

**Keywords:** rhinovirus, air-liquid interface, airway epithelium, IL-17C, well-differentiated, basolateral secretion

## Abstract

Human rhinovirus (HRV) is a major trigger of acute exacerbations of both asthma and chronic obstructive pulmonary disease. The airway epithelium is the primary site of HRV infection, and responds by releasing proinflammatory and antimicrobial cytokines. Epithelial cells release IL-17C in response to exposure to bacterial, viral, and fungal pathogens. We previously demonstrated a role for HRV in IL-17C production from undifferentiated epithelial cells, and showed that IL-17C could play a role in neutrophil recruitment. To extend these observations, highly differentiated human bronchial epithelial cells (HBE) were infected apically with HRV to assess the effect of dose, time, viral replication, and strain on the IL-17C response. Cellular lysates, and basolateral and apical secretions were analyzed for IL-17C and CXCL1 protein release following HRV or IL-17C stimulation. Upon HRV infection, IL-17C protein was exclusively released basolaterally in a dose-, time-, and viral replication-dependent manner. Several strains of rhinovirus were capable of inducing IL-17C release. Enriched columnar epithelial cell populations contained significantly higher viral titer, and expressed significantly more IL-17C mRNA than enriched basal cell populations. In addition, the kinetic profile of IL-17C release following HRV treatment closely mimics viral shedding kinetics, further implicating the role of rhinovirus replication in IL-17C production. Basolateral treatment of HBEs with IL-17C resulted in a dose-dependent increase in basolateral CXCL1 production. In summary, replicating rhinovirus drives basolateral IL-17C protein release from both apical and basal epithelial cells, which may then act in an autocrine/paracrine manner to promote basolateral CXCL1 protein release.

## Introduction

Human rhinovirus (HRV) is the most common respiratory virus associated with exacerbations of asthma and chronic obstructive pulmonary disease patients (Leigh and Proud, 2015). Airway epithelial cells are the primary site of HRV infection and replication, and such infections induce epithelial production of numerous chemical mediators, including antivirals that limit infection, but also cytokines and chemokines that contribute to airway inflammation and to exacerbations of lower airway diseases (Leigh and Proud, 2015).

IL-17C is a member of the IL-17 cytokine family produced by epithelial cells, and is induced in response to bacterial (Ramirez-Carrozzi et al., 2011; Song et al., 2011; Pfeifer et al., 2013; Roth et al., 2014; Wolf et al., 2016; Steck et al., 2019; Jeon et al., 2020), viral (Ioannidis et al., 2012; Peng et al., 2017), or fungal infections (Conti et al., 2015; Huang et al., 2016). In the lung, several studies have shown that bacteria can induce IL-17C in cell culture systems and in animal models (Ramirez-Carrozzi et al., 2011; Pfeifer et al., 2013; Wolf et al., 2016). In the context of respiratory viral infections, one study observed that influenza virus, but not respiratory syncytial virus, induced IL-17C gene expression in human and murine bronchial epithelial cells at air-liquid interface (ALI) (Ioannidis et al., 2012). Increased IL-17C expression in response to influenza was attenuated, but not abolished, in murine bronchial epithelial cells from IFNAR-/- and STAT-/- mice (Ioannidis et al., 2012). We have shown that rhinovirus synergistically induced IL-17C gene and protein expression in undifferentiated primary human bronchial epithelial cells when treated concurrently with bacteria (Jamieson et al., 2019). Induction of IL-17C following rhinovirus-bacteria co-exposure required viral replication and was dependent on RIG-I and MDA5, two cytosolic viral replication recognition sensors, as well as on NF-κB and p38 signaling (Jamieson et al., 2019).

IL-17C acts on a heterodimeric receptor comprising IL-17RA and IL-17RE, where IL-17RE is the selective subunit (Chang et al., 2011; Ramirez-Carrozzi et al., 2011; Song et al., 2011). Gene expression studies have found IL-17RE to be primarily located on epithelial cells in mucosal tissues including the lung, trachea, mouth, stomach, and colon (Li et al., 2006; Ramirez-Carrozzi et al., 2011). Previous studies in murine and *in vitro* models have shown that IL-17C acts on the epithelium in an autocrine/paracrine manner to induce CXCL1 release and neutrophil recruitment (Wolf et al., 2016; Jamieson et al., 2019; Steck et al., 2019). But this has not yet been examined in highly differentiated HBE.

To gain additional insights into the control and vectoriality of IL-17C production, as well as to study potential autocrine paracrine responses to IL-17C in cells that more closely resemble an *in vivo* airway epithelium, we used highly differentiated HBE grown at air-liquid interface. We initially hypothesized that IL-17C would be released both apically and basolaterally from HRV-infected highly differentiated HBE, and that release would require HRV replication. We also determined whether apical or basolateral stimulation with exogenous IL-17C would induce subsequent CXCL1 production.

## Materials and Methods

### Bronchial Epithelial Cell Cultures

Primary HBE were obtained from non-transplanted human lungs from normal donors via a tissue retrieval service (International Institute for the Advancement of Medicine, Edison, NJ). Ethical approval to obtain HBE was obtained from the Conjoint Health Research Ethics Board of the University of Calgary (Calgary, AB, Canada) and from the Internal Ethics Board of the International Institute for the Advancement of Medicine. For the current work, a total of 18 different lung donors were used (age range 13–63 years; 11 male/7 female). All subjects died of either cerebrovascular disease or from head trauma. Primary human bronchial epithelial (HBE) cells were obtained by protease digestion of dissected airways (main stem bronchus to 4th generation) as previously described (Churchill et al., 1989).

HBE cells were cultured on T75 cm^2^ flasks (Costar, Corning Inc., Corning, NY) in Bronchial Epithelial Growth Medium (BEGM, Lonza, Walkersville, MD) supplemented with 5% FBS for 72 h (Life Technologies, Burlington, Ontario, Canada). Cells were then fed every 48 h with BEGM without FBS. At 90% confluence, cells were lifted and seeded at 2.0 × 10^5^ cells per insert onto 1.12 cm^2^, 0.4 μm pore transwell inserts (Costar) coated with bovine collagen Type I/III (Advanced BioMatrix, San Diego, CA), and cultured in BEGM for 48 h. BEGM was then removed and HBE were cultured using only basolateral PneumaCult-ALI differentiation medium containing 100X supplement, hydrocortisone, and heparin (Stemcell Technologies, Vancouver, BC, Canada), as well as fluconazole (Sigma-Aldrich, Oakville, Ontario, Canada) and penicillin/streptomycin (Life Technologies). Cells were fed basolaterally every 48 h. Beginning 14 days after seeding, cells were washed apically once per week with PBS to remove excess mucus. Cultures were used for experiments at 5 weeks after transwell seeding, as previously described (Warner et al., 2019). Each n value represents the use of a distinct epithelial cell donor.

### Purified Rhinovirus Stocks

Stocks of HRV-16 were propagated in WI-38 fibroblasts, while HRV-1A was propagated in H1-HeLa cells. Both viruses were purified via centrifugation over a sucrose cushion as previously described and titered in the same cell lines used for propagation, as previously described (Sanders et al., 1998; Shelfoon et al., 2016; Maciejewski et al., 2017). Replication deficient viruses were produced by exposure for 5 min to a Spectroline Model XX-15F high intensity short wavelength (254 nm) UV lamp (Spectronics Corp., Westbury, NY) at a distance of 5 cm. Inactivation of viral replication was confirmed by showing a failure to replicate in appropriate fibroblast host cells. We have previously shown that this brief UV treatment does not prevent interaction of HRV with its receptor or triggering of early, replication-independent signaling (Wang et al., 2006).

A cDNA plasmid encoding for HRV-C15 was a generous gift from Drs. Yuri Bochkov and James Gern (University of Wisconsin). Infectious HRV-C15 stocks were produced by reverse transcription and transfection into WI-38 cells as previously described (Bochkov et al., 2011). Following purification, sucrose was removed from virus stocks by dialysis.

### Epithelial Cell Stimulation

All experiments were performed in complete PneumaCult ALI medium supplemented with 1% Penicillin/Streptomycin and 0.5% Fluconazole. The apical surface was exposed to HRV in 100 μl of F12 with 25 mM HEPES for 4 h. Cells were washed 5X with PBS to remove residual HRV used for infection. The first and final washes were analyzed for viral RNA to ensure successful removal of HRV. Fresh basolateral medium (1 ml) was added to each well and this media was collected at appropriate times to analyze basolateral secretions. Apical secretion of virus or mediators was assessed at appropriate time points by rinsing the apical surface of ALI cultures with 500 μL PBS.

### RNA Extraction, cDNA Conversion and Real-Time PCR

Total cellular or viral RNA were isolated as previously described (Warner et al., 2019). Real-time RT-PCR quantification of IL-17C, IL-17RA, IL-17RE, and HRV were performed using specific primers and a TaqMan probe and expressed in absolute quantities using a first strand cDNA standard curve. HRV levels were expressed as copy number, with each copy number representing a viral genome. For all other genes, specific primers were used and samples were quantified using the 2^−ΔΔ*CT*^ method (Livak and Schmittgen, 2001), and standardized to the housekeeping gene, GAPDH. IL-17C primer and probe sequences were as previously described (Jamieson et al., 2019).

### IL-17C and CXCL1 ELISA Duosets

IL-17C and CXCL1 protein levels were measured by ELISA using duosets provided by the manufacturer (R&D Systems, Minneapolis, MN). Recombinant human IL-17C was from R&D Systems (Catalog# 1234-IL-025).

### Air-Liquid Interface Trypsin Cell Separation

Separation of columnar and basal cell layers involved 5 apical washes, followed by incubation with 0.025% trypsin in PBS in apical and basolateral compartments for 15–20 min at 37°C. “Columnar cells” were removed and collected by forceful jetting of the apical surface. The remaining cells were washed with PBS and incubated with 0.025% trypsin in PBS for 10 min at 37°C. “Basal cells” were removed by jetting. Apical and basal cell populations were processed for RNA isolation and analysis.

### Histology

HBE were fixed in 10% neutral buffered formalin, embedded in paraffin, and sectioned to 4 μm thickness onto Superfrost plus slides. Alcian blue and hematoxylin staining was performed by de-paraffinization in two changes of xylene and rehydrating through graded ethanol solutions (100, 95, 70% EtOH). Alcian blue 8GX (Sigma) in 3% acetic acid solution was added for 2 min and rinsed in water for 2 min. Hematoxylin Gills II (Leica Biosystems) staining was performed for 5 min and rinsed in warm tap water for 5 min. Slides were dehydrated through reverse graded ethanol solutions (95, 100% EtOH) and cleared in two changes of xylene before applying coverslip with Permount (Thermofisher). Images were captured using an Olympus BX51 microscope using a 40X objective and Q Capture Pro 6.0 software.

### Statistical Analysis

Normality was assessed using the Kolmogorov-Smirnov test. Parametric data were analyzed using appropriate one-way or two-way ANOVA with Holm-Sidak *post-hoc* analysis, as appropriate. Non-parametric data were analyzed using a Kruskal-Wallis test with a Dunn's *post-hoc* analysis. All statistical tests were performed using GraphPad Prism 7 (GraphPad Software, Inc., La Jolla, CA), and *p* < 0.05 were considered significant (^*^p < 0.05; ^**^p < 0.01; ^***^p < 0.001).

## Results

### HRV Induces Basolateral IL-17C Release in a Dose-Dependent and Replication-Dependent Manner

**Figure 1:**
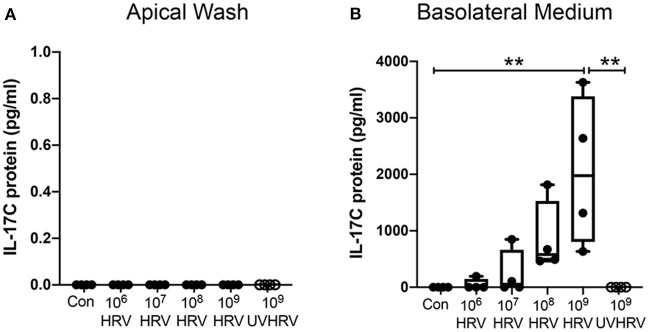
IL-17C is released basolaterally in response to HRV-1A in a dose- and replication-dependent manner. Highly differentiated HBE were treated with control medium (Con), with increasing doses of HRV-1A (10^6^-10^9^ HRV copies) or 10^9^ copies of replication-deficient HRV-1A (UVHRV) for 4 h. **(A)** Apical wash and **(B)** basolateral medium were collected at the time of harvest and analyzed for IL-17C release (*n* = 4). Significant differences were assessed using a Kruskal-Wallis ANOVA with Dunn's multiple comparisons *post-hoc* test and indicated with asterisks. **p < 0.01.

Highly differentiated HBE were treated apically with increasing doses of HRV-1A. IL-17C protein was not constitutively released but HRV-1A induced basolateral secretion of IL-17C in a dose-dependent manner (Figure 1B). Replication deficient (UV-treated) HRV-1A (at the highest dose used for live virus) did not induce basolateral release of IL-17C (Figure 1B). Apical IL-17C release was not detected in response to HRV-1A (Figure 1A).

### IL-17C Protein Release Peaks Between 12 and 48 h Following HRV Infection

**Figure 2:**
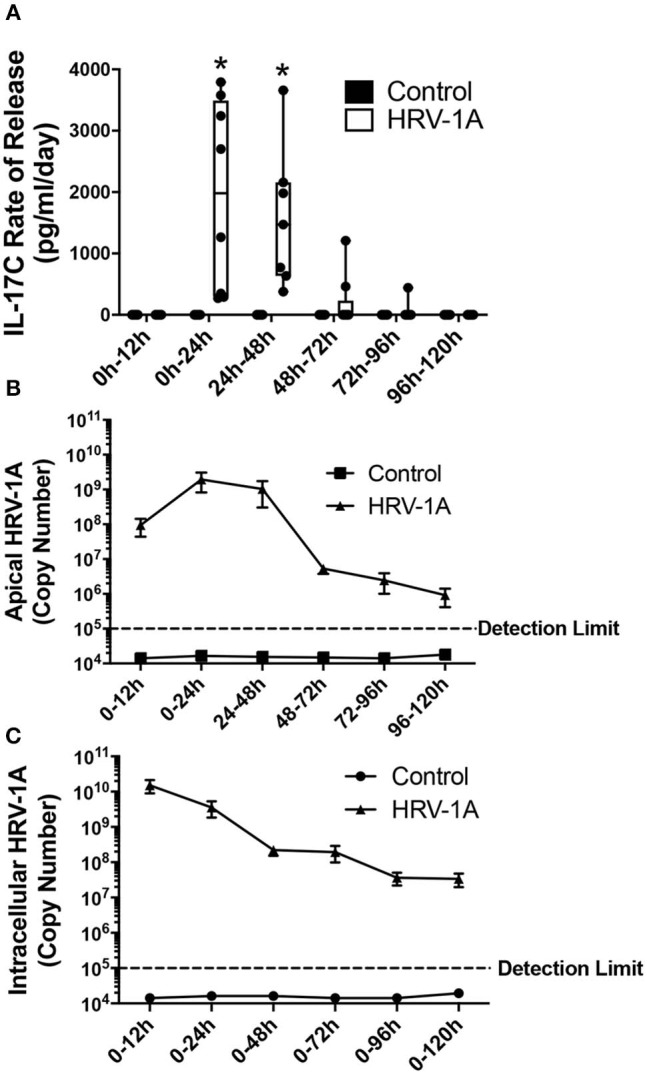
The daily rate of basolateral IL-17C release peaks between 12 and 48 h following HRV-1A exposure. Highly differentiated HBE were treated apically with 10^8^ copy number HRV-1A for 12, 24, 48, 72, 96, or 120 h and apical wash and basolateral medium were collected every 24 h. Cellular RNA was collected at each time point and represents cumulative levels in the presence of daily apical washes and basolateral medium changes (*n* = 6–8). **(A)** IL-17C protein was measured via ELISA in the basolateral medium collected following indicated time periods. **(B)** Rhinovirus RNA was measured via real-time RT-PCR in the apical washes collected following indicated time periods. **(C)** Intracellular rhinovirus RNA was a cumulative measurement from *t* = 0 after daily washes and medium changes, and was measured via real-time RT-PCR in cellular lysates. Significant differences were assessed using a Two-way ANOVA with Holm-Sidak's multiple comparisons test at each time point and indicated with asterisks. *p < 0.05. The detection limit indicates the minimum viral copy number detected with real-time RT-PCR.

**Figure 3:**
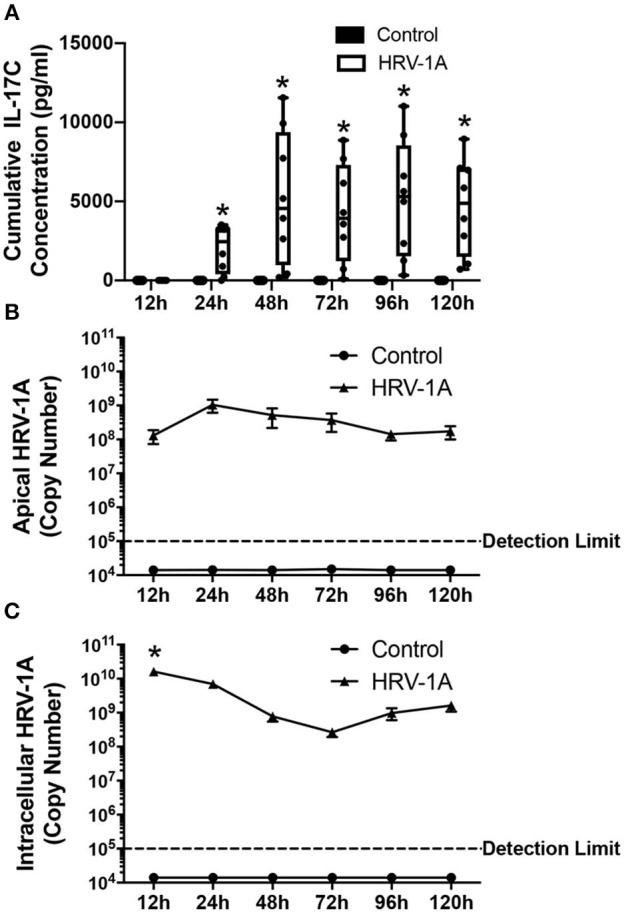
Cumulative basolateral IL-17C release remains significantly induced from 24 to at least 120 h following HRV-1A exposure. Highly differentiated HBE were treated apically with 10^8^ copy number HRV-1A for 12, 24, 48, 72, 96, or 120 h and apical wash, basolateral medium, and intracellular RNA was collected at time of harvest (*n* = 6–8). **(A)** IL-17C protein was measured via ELISA in the basolateral medium. **(B)** Apically shed RNA and **(C)** cellular RNA were isolated and analyzed for HRV-1A copy number. Significant differences were assessed using a Two-way ANOVA with Holm-Sidak's multiple comparisons test at each time point and indicated with asterisks. *p < 0.05. The detection limit indicates the minimum viral copy number detected with real-time RT-PCR.

The kinetic profile of IL-17C release following HRV-1A infection was compared to levels of both apically shed and intracellular HRV genomic RNA. The daily amount of IL-17C released basolaterally, or of the amount of virus shed apically, within each 24-h period were assessed in basolateral medium and apical washes collected each day. Cumulative intracellular HRV RNA levels were also assessed at each time point following daily washes and basolateral medium changes. No significant basolateral release of IL-17C protein was observed between 0 and 12 h after HRV exposure. However, significant basolateral IL-17C production was detected between 0 and 24 h following HRV treatment, and between 24 and 48 h following HRV treatment (Figure 2A). At all time points later than 48 h, basolateral IL-17C levels were not significantly different than control (Figure 2A). Apical shedding of HRV was detectable by 12 h and showed peak production between 0 and 24 h, and 24–48 h before declining sharply (Figure 2B). By comparison, intracellular HRV appeared to peak between 0 and 12 h post-stimulation and then gradually declined (Figure 2C). We also monitored cumulative rates of IL-17C release and of intracellular and apically shed HRV. For these measurements, samples were measured only at the time point assessed, with no removal of medium at any earlier time point. Significant cumulative production of IL-17C protein was detected at 24 h, and was further increased or maintained for up to 120 h (Figure 3A). Both apically shed and intracellular HRV were induced by 12 h and levels were maintained up until 120 h post-infection (Figures 3B,C). No detectable levels of IL-17C or HRV were observed under control (medium stimulation) conditions.

### Basolateral IL-17C Protein Induction Is Not Rhinovirus Species-, or Receptor-Specific

**Figure 4:**
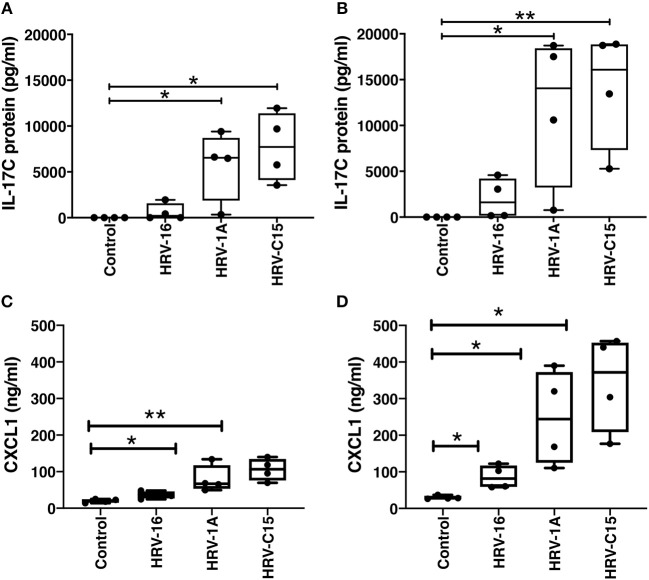
Basolateral IL-17C release can be induced in response to multiple serotypes of human rhinovirus as can CXCL1. Highly differentiated HBE were treated apically with major group virus HRV-16 (10^8^ copies), minor group virus HRV-1A (10^8^ copies), or C clade virus HRV-C15 (10^9^ copies) for **(A,C)** 24 h or **(B,D)** 48 h and basolateral media were collected at the time of harvest (*n* = 4). Basolateral media were analyzed for release of IL-17C **(A,B)** and CXCL1 **(C,D)**. Significant differences were assessed using a Kruskal-Wallis ANOVA with Dunnett's multiple comparisons *post-hoc* test and indicated with asterisks. *p < 0.05, **p < 0.01.

Basolateral IL-17C protein release was not selective for a specific rhinovirus receptor. Significant basolateral release of IL-17C was observed at both 24 and 48 h following apical treatment not only with HRV-1A (10^8^ copies), which uses the LDL-receptor family for binding and cell entry (Hofer et al., 1994), but also with HRV-C15 (10^9^ copies), which uses cadherin related family member 3 (CDHR3) to gain cell entry (Bochkov et al., 2015). HRV-16 (10^8^ copies), which uses ICAM-1 as its receptor (Greve et al., 1989), led to measurable levels of IL-17C release from all 4 donors at 48 h post infection, but these were not significantly increased compared to control because of the wide range of individual values (Figures 4A,B). The synthetic double-stranded RNA mimic, Poly I:C also induced IL-17C in a dose-, and time-dependent manner (data not shown). Because we have previously shown that IL-17C can feedback on epithelial cells to induce release of the neutrophil chemoattractant, CXCL1 (Jamieson et al., 2019), we also monitored levels of this chemokine in the same samples used to measure IL-17C. The pattern of CXCL-1 production by different rhinovirus strains mirrored that seen for IL-17C. At 24 h post infection, significant production of CXCL1 was seen with both HRV-1A and HRV-C15, while at 48 h post infection, all 3 strains of HRV induced significant production of CXCL1 (Figures 4C,D).

### Columnar Cells Are the Major Site of HRV1A Infection and IL-17C Expression

**Figure 5:**
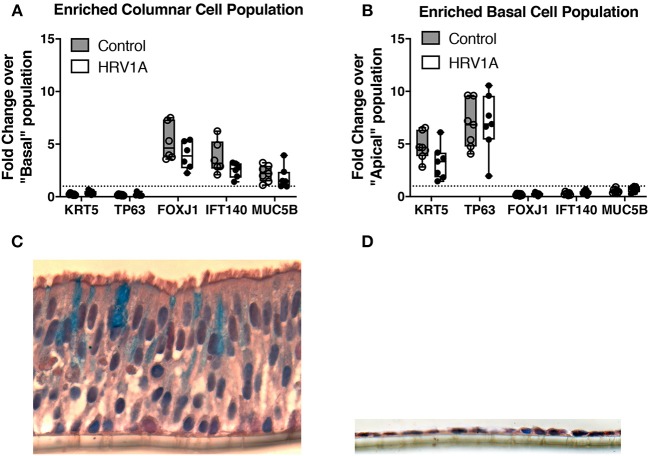
Characterization of trypsin-separated columnar and basal cell enriched populations from highly differentiated cultures of HBE. Highly differentiated HBE were treated with 0.025% trypsin in the apical and basolateral compartment for 20 min at 37°C. The columnar layer was jetted off the insert and collected for RNA isolation. The remaining basal layer was treated with 0.025% trypsin in the apical and basolateral compartments for 10 min at 37°C. Cells from the basal layer were collected separately (*n* = 7). **(A,B)** RNA from each subpopulation were isolated and analyzed for markers of columnar cells (FOXJ1/IFT140/MUC5B) and basal cells (KRT5/TP63). Horizontal dashed line represents a value of 1 where genes would be equally distributed between populations. **(C,D)** Cells were imaged before and after trypsin-separation and stained with alcian blue and haematoxylin counterstain.

**Figure 6:**
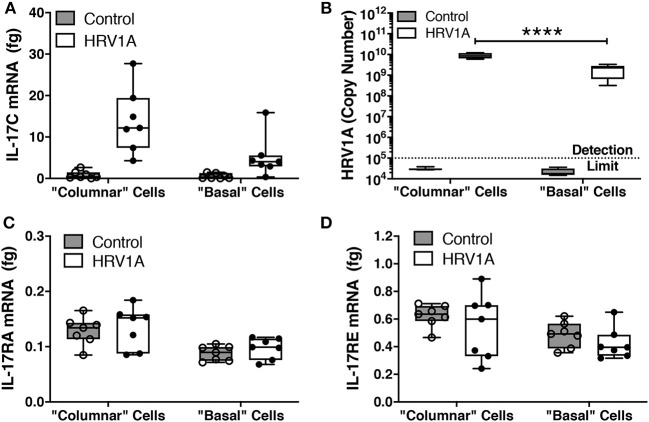
Expression of mRNA for IL-17C, IL-17RA, and IL-17RE, and HRV1A genomic RNA in columnar and basal cell enriched populations. Highly differentiated HBE were infected with 10^8^ copy number of HRV-1A for 24 h. Cell fractions enriched for columnar and basal cell populations were isolated as described in the legend for Figure 5 (*n* = 7). RNA from each subpopulation was isolated and analyzed for **(A)** IL-17C, **(B)** HRV-1A, **(C)** IL-17RA, and **(D)** IL-17RE. Significant differences were assessed using a Two-way ANOVA with Holm-Sidak's multiple comparisons *post-hoc* test comparing HRV-1A between cell populations and indicated with asterisks. ****p < 0.0001.

Following trypsin separation of the columnar and basal cell populations from differentiated HBE, enrichment of appropriate cell populations was confirmed by analyzing expression of basal cell gene markers (KRT5 and TP63), ciliated cell gene indicators (FOXJ1 and IFT140) and of the goblet cell marker, MUC5B (Figures 5A,B). The successful removal of columnar cells was also confirmed by histology (Figures 5C,D). Expression levels of IL-17C, HRV-1A, IL-17RA, and IL-17RE mRNA were also assessed in columnar and basal cell populations. When equal quantities of total cellular RNA from both columnar and basal cell populations were compared, the enriched columnar cell population showed significantly increased IL-17C, HRV-1A, and IL-17RA gene expression compared to the enriched basal cell population (Figures 6A–C). By contrast, expression of the selective IL-17C receptor, IL-17RE, was not significantly different between the two enriched cell populations (Figure 6D). Interestingly, greater absolute levels of IL-17RE mRNA was detected compared to IL-17RA mRNA, but HRV infection did not alter expression levels of either receptor subunit (Figures 6C,D).

### Apical IL-17C Stimulation Does Not Induce CXCL1 Release, While Basolateral IL-17C Stimulation Induces Dose-Dependent Basolateral CXCL1 Release

**Figure 7:**
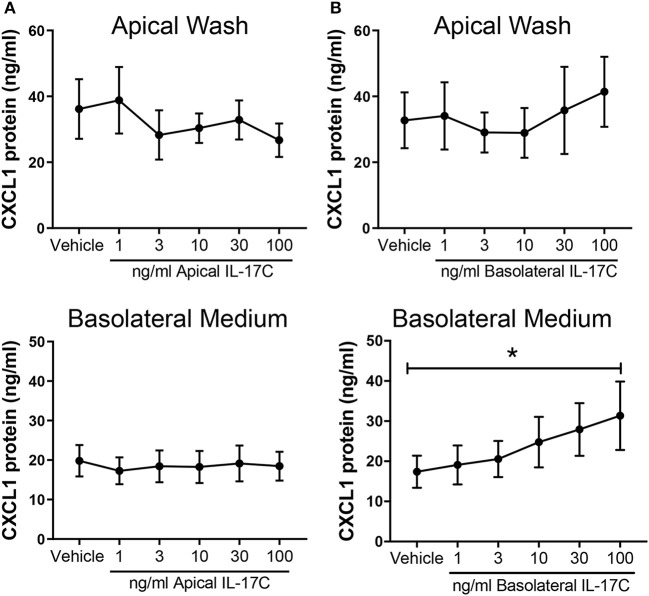
IL-17C acts in a concentration-dependent manner at the basolateral surface to induce basolateral release of CXCL1. Highly differentiated HBE were treated **(A)** apically or **(B)** basolaterally with increasing doses of exogenous IL-17C (1–100 ng/ml) for 24 h (*n* = 6). Apical washes and basolateral media were collected at time of harvest and analyzed for CXCL1 release. Basolateral generation of CXCL1 in response to 100 ng/ml of IL-17C applied basolaterally was significantly different than control. *p < 0.05.

HBE were treated with increasing doses of IL-17C apically or basolaterally for 24 h. Apical stimulation with IL-17C had no detectable effect on apical or basolateral release of CXCL1 (Figure 7A). By contrast, basolateral treatment with IL-17C had no effect on apical levels of CXCL1, but induced a dose-dependent release of CXCL1 into basolateral medium that was significantly increased above control in response to 100 ng/ml of IL-17C (Figure 7B).

## Discussion

Interleukin 17C is a member of the IL-17 family of cytokines that is expressed in airway epithelial cells. Increased epithelial expression of IL-17C has been observed in response to a variety of bacterial, viral, and fungal infections (Ramirez-Carrozzi et al., 2011; Song et al., 2011; Ioannidis et al., 2012; Pfeifer et al., 2013; Huang et al., 2016; Jamieson et al., 2019), but the role of IL-17C appears variable, as both protective (Ramirez-Carrozzi et al., 2011; Song et al., 2011; Kusagaya et al., 2014) and pathogenic (Ramirez-Carrozzi et al., 2011; Huang et al., 2016) functions have been reported in various bacterial or fungal infections. Previous *in vitro* studies using undifferentiated cells, as well as *in vivo* mouse studies have provided some insights into the pathways involved in regulation of IL-17C expression, but such models have limitations. We chose to examine highly differentiated cultures of HBE that recapitulate an *in vivo* architecture, as such cultures provide a unique opportunity to assess the longer-term kinetics of protein release of IL-17C, as well as the vectorial nature of protein release and responses.

Although it was necessary to use HRV in combination with bacteria to generate measurable levels of IL-17C from submersion cultures of HBE (Jamieson et al., 2019), we found that HRV alone was capable of independently inducing robust IL-17C protein release from highly differentiated cultures of HBE. As such, we focused on characterizing responses to HRV alone in these studies. HRV-1A induced basolateral IL-17C release in a dose dependent manner. Surprisingly, IL-17C protein was not detected in the apical wash samples but was consistently measured in the basolateral medium following HRV infection. This may have implications for *in vivo* studies, as the polarized basolateral release of IL-17C could mean that IL-17C, in contrast to IL-17A and IL-17E (Southworth et al., 2019), may be difficult to detect in airway surface secretions recovered by lavage or aspiration. This vectorial distribution of IL-17C is not a generalized phenomenon, as other cytokines induced by HRV, including IFN-λ, CXCL10, and HBD2, could be detected in both the apical and basolateral secretions (Warner et al., 2019) (and data not shown), although levels were not necessarily equivalent in both directions. There could be several non-mutually exclusive explanations for this observed vectoriality of IL-17C release. It is conceivable that only basal cells produce IL-17C leading to basolateral release, or that there is a directional sorting system that selectively leads to basolateral release of IL-17C. Alternatively, it may be that apical secretion of IL-17C occurs but at levels less than the limit of detection of the assay used. Finally, it is possible that IL-17C is released into the apical fluid but is degraded by surface bound or secreted proteases that are not present at the basolateral surface.

The detection limit on the ELISA used for IL-17C is ~75 pg/ml, and assays with significantly greater sensitivity are not currently available. If apical IL-17C is generated at levels below detection, it would still imply that apical secretion is at least some 20-fold below that regularly detected in basolateral fluids. To assess the potential role of proteolytic degradation at the apical surface we examined degradation of exogenous IL-17C added to the apical surface alone or in the presence of a protease inhibitor cocktail that should inhibit serine, cysteine, and aspartic proteases, as well as aminopeptidases. Over the course of 24 h, less exogenous IL-17C was detectable with time and IL-17C levels were significantly higher when the protease inhibitor cocktail was present, however IL-17C remained detectable for the duration of the experiment (data not shown). This suggested that while proteases may contribute to degradation of IL-17C with time, it should still be detectable in apical secretions. A limitation of these studies is that available inhibitors of metalloproteinases could not be used in the inhibitor mix as they impacted epithelial integrity by chelating ions.

To determine whether apical or basal cells were predominantly expressing IL-17C following HRV infection, we enriched columnar and basal cell fractions using differential trypsin digestion. Logically, columnar or apical cells should have greater exposure to an apical HRV infection than basal cells. Consistent with this, we detected significantly more HRV RNA in the apical cell fraction compared to the basal cell fraction. Although levels in columnar cells were at least one log higher than in basal cells, the detection of viral genome in basal cells implies that cell-to-cell spreading of infection can occur. IL-17C mRNA expression showed a similar trend, with columnar cells expressing significantly more IL-17C than basal cells following apical HRV exposure, confirming that cells infected with HRV were releasing IL-17C. Given that columnar cells are predominantly involved in IL-17C expression, it suggests that intracellular IL-17C is specifically packaged and sorted toward the basolateral surface. Basolateral protein trafficking is typically associated with basolateral sorting signals, such as mono- and di-leucine residues, and such signals exist within the cytosol-facing domain of the IL-17C protein sequence (Stoops and Caplan, 2014). Future studies using high resolution live imaging with suitable, highly specific reagents to visually assess IL-17C production and release in real-time would be required to validate this hypothesis.

In our previous study using HBE grown in submersion culture, IL-17C was not constitutively produced, and protein was not detectable until 18 h post stimulation with pathogens (Jamieson et al., 2019). Our current studies using highly differentiated cells were consistent with these earlier studies, also showing no constitutive production of IL-17C, nor detection over the first 12 h after HRV exposure, but with significant basolateral IL-17C protein release consistently detected at 24 h post-infection. During cumulative measurements, HRV-induced cytokines were allowed to accumulate in the apical and basolateral milieu for increasing time periods, and IL-17C protein levels in the basolateral medium appeared to peak around 48 h post infection. After this point, high IL-17C levels were consistently measured up to 120 h post-infection. This sustained kinetic response could be a result of early protein release and stable expression in the basolateral compartment, or a result of continued IL-17C release, due to consistent virus exposure and simultaneous IL-17C protein degradation. To differentiate between these two possibilities we also assessed the daily rate of IL-17C release and apical HRV shedding to determine if IL-17C was continuously released over time. This daily analysis of protein kinetics revealed that IL-17C protein release predominantly occurred between 12 and 48 h and was no longer consistently released after 48 h, suggesting either that (1) early release of IL-17C persists without significant degradation up to 120 h post-infection, and/or (2) that the reduction in apically shed HRV due to daily washes also limits further infection of airway epithelial cells contributing to the reduction in IL-17C. While there are limitations to both time course models, there is a clear association with viral shedding and basolateral IL-17C release.

The importance of viral replication in subsequent IL-17C induction is further demonstrated when the treatment with a high dose of HRV rendered replication-deficient by UV-treatment did not induce IL-17C release. Several studies have implicated the viral replication by-product, double-stranded RNA, with IL-17C gene and protein expression, as well as the cellular pathogen recognition receptors RIG-I and MDA5 (Pfeifer et al., 2013; Kusagaya et al., 2014; Jamieson et al., 2019). Thus far, influenza, herpes simplex virus (HSV), and HRV have been shown to induce IL-17C gene and/or protein expression (Ioannidis et al., 2012; Peng et al., 2017; Jamieson et al., 2019). Moreover, our current data demonstrate that both HRV-16 and HRV-1A are able to induce IL-17C protein expression. These are both members of the HRV-A clade but they interact with distinct cellular receptors (Jamieson et al., 2019). Our current study is the first to show that, in addition to HRV-A species, HRV-C15, which uses CDHR3 to gain cell entry (Bochkov et al., 2015), also is capable of inducing significant IL-17C protein release at 24 and 48 h. It should be noted that the purpose of these studies was not to quantitatively compare responses to different HRV strains but solely to demonstrate that multiple strains using differing receptors induced IL-17C production. Because we have previously shown that IL-17C can feedback on epithelial cells to induce release of the neutrophil chemoattractant, CXCL1 (Jamieson et al., 2019), we also monitored levels of this chemokine in the same samples used to measure IL-17C. The pattern of CXCL-1 production by different rhinovirus strains mirrored that seen for IL-17C. Although this is consistent with a contribution of IL-17C in the production of CXCL1, caution must be taken in interpretation of this data, as it may also reflect similar induction pathways of both cytokines in response to HRV infection.

In contrast to our data showing that induction of IL-17C by HRV requires viral replication, it has been reported that Herpes Simplex Virus (HSV)-2-induced IL-17C gene expression levels in keratinocytes were not affected by UV-treatment of HSV or by treatment with the anti-viral acyclovir, suggesting that HSV replication may not contribute to the IL-17C response in human keratinocytes (Peng et al., 2017). These differences may reflect different virus structures as HSV-2 is an enveloped DNA virus that may trigger effects either directly via interaction of envelope surface glycoproteins with 3-O-sulfated heparan sulfate or via events subsequent to the interaction of its gD glycoprotein with any of at least 3 surface receptors (Clarke, 2015).

The exclusive basolateral IL-17C secretion observed from differentiated HBE implies that any potential autocrine/paracrine effects of this cytokine would require that it interacts with receptors on the basolateral surface. We assessed if both columnar and basal cells express genes for the heterodimeric components of the IL-17C receptor, IL-17RA and IL-17RE. IL-17RA is known to be constitutively expressed on most cell types including lung epithelial cells (Chang and Dong, 2011), while IL-17RE is the selective subunit and is primarily localized on epithelial cells in the mouth, stomach, lung, trachea, skin, and kidney (Ramirez-Carrozzi et al., 2011; Al-Samadi et al., 2014; Huang et al., 2016), as well as on Th17 cells (Chang and Dong, 2011), dermal and colonic fibroblasts (Ramirez-Carrozzi et al., 2011), and nerve endings (Peng et al., 2017). We examined mRNA expression of both the IL-17RA and IL-17RE receptor subunits in columnar and basal cell enriched fractions, at baseline and following infection with HRV. We found that mRNA for both subunits was constitutively expressed in both columnar and basal cells and was not increased in either cell population after HRV infection. Significantly more IL-17RA mRNA was detected in columnar cells than in basal cells. Although a similar trend was observed for IL-17RE, this was not statistically significant. A significant limitation of these observations is that examining mRNA expression does not provide information on the localization or level of receptor protein expression. Thus, even though mRNA may be expressed in both cell types, the relative distribution of apical compared to basolateral receptor protein expression remains unclear. Given that, in a pseudostratified airway epithelium, both columnar and basal cells contact the basement membrane, receptors in both cell types could conceivably be dominantly expressed on basolateral membrane domains. Although we considered imaging studies to evaluate relative potential distributions, available antibodies, particularly to IL-17RE, detected multiple bands on western blots, suggesting that any staining obtained could be misleading or not interpretable.

We had previously shown in HBE grown in submersion culture that IL-17C release from epithelial cells contributed to neutrophil chemotaxis via autocrine/paracrine induction of chemokines including CXCL1 (Jamieson et al., 2019). Thus, as an alternative approach to assess receptor distribution, we examined the effects of exogenous IL-17C applied either apically or basolaterally on release of CXCL1 from highly differentiated HBE. Apical administration of IL-17C caused no significant increases above baseline in CXCL1 production into either apical or basolateral secretions, implying that few receptors must exist on the apical surface. By contrast basolateral treatment with IL-17C caused a concentration-dependent increase in the basolateral, but not apical, release of CXCL1, with levels observed using 100 ng/ml IL-17C being significantly increased compared to control. These data would support the concept that receptors for IL-17C are predominantly expressed on the basolateral surface of differentiated epithelial cells.

In summary, therefore, we demonstrate that apical infection of airway epithelial cells with HRV induces a vectorial basolateral IL-17C protein release, likely from both apical and basal HBE cells. Following basolateral release, IL-17C interacts with receptors located on the basolateral surface in an autocrine/paracrine manner, to induce basolateral CXCL1 protein release, which, in turn may contribute to exacerbations of lower airway disease via recruitment of increased numbers of neutrophils to the airway surface.

## Data Availability Statement

All datasets generated for this study are included in the article/supplementary material.

## Ethics Statement

The studies involving human participants were reviewed and approved by Conjoint Health Research Ethics Board of the University of Calgary. The patients/participants provided their written informed consent to participate in this study.

## Author Contributions

KJ and DP conceptualized and designed the study. KJ, SW, and AM worked on the data collection. KJ and DP analyzed the data. KJ, SW, AM, and DP prepared the manuscript.

### Conflict of Interest

The authors declare that the research was conducted in the absence of any commercial or financial relationships that could be construed as a potential conflict of interest.
